# AGR2, a unique tumor-associated antigen, is a promising candidate for antibody targeting

**DOI:** 10.18632/oncotarget.26945

**Published:** 2019-07-02

**Authors:** Alvin Y. Liu, Adelle D. Kanan, Tomasz P. Radon, Siama Shah, Mark E. Weeks, Julie M. Foster, Jane K. Sosabowski, Laurent Dumartin, Tatjana Crnogorac-Jurcevic

**Affiliations:** ^1^ Department of Urology, Institute for Stem Cell and Regenerative Medicine, University of Washington, Seattle, WA, USA; ^2^ Centre for Molecular Oncology, Barts Cancer Institute, Queen Mary University of London, London, UK

**Keywords:** pancreatic cancer, eAGR2, chimeric antibody, prostate cancer, tumor localization

## Abstract

Anterior gradient 2 (AGR2), a protein disulfide isomerase, shows two subcellular localizations: intracellular (iAGR2) and extracellular (eAGR2). In healthy cells that express AGR2, the predominant form is iAGR2, which resides in the endoplasmic reticulum. In contrast, cancer cells secrete and express eAGR2 on the cell surface. We wanted to test if AGR2 is a cancer-specific tumor-associated antigen. We utilized two AGR2 antibodies, P3A5 and P1G4, for *in vivo* tumor localization and tumor growth inhibition. The monoclonal antibodies recognized both human AGR2 and mouse Agr2. Biodistribution experiments using a syngeneic mouse model showed high uptake of P3A5 AGR2 antibody in xenografted eAgr2^+^ pancreatic tumors, with limited uptake in normal tissues. In implanted human patient-derived eAGR2^+^ pancreatic cancer xenografts, tumor growth inhibition was evaluated with antibodies and Gemcitabine (Gem). Inhibition was more potent by P1G4 + Gem combination than Gem alone or P3A5 + Gem. We converted these two antibodies to human:mouse chimeric forms: the constructed P3A5 and P1G4 chimeric mV_L_hC_κ_ and mV_H_hC_γ_ (γ1, γ2, γ4) genes were inserted in a single mammalian expression plasmid vector, and transfected into human 293F cells. Expressed human:mouse chimeric IgG1, IgG2 and IgG4 antibodies retained AGR2 binding. Increase in IgG yield by transfected cells could be obtained with serial transfection of vectors with different drug resistance. These chimeric antibodies, when incubated with human blood, effectively lysed eAGR2^+^ PC3 prostate cancer cells. We have, thus, produced humanized anti-AGR2 antibodies that, after further testing, might be suitable for treatment against a variety of eAGR2^+^ solid tumors.

## INTRODUCTION

Based on current figures, it is estimated that by 2040 there will be close to 30 million new cancer cases worldwide (http://gco.iarc.fr/tomorrow/home). Solid tumors are by far the most common, including lung, breast and prostate cancers. Pancreatic cancer, incidence of which is increasing, is particularly aggressive with a relatively short span between diagnosis and death.

AGR2 (anterior gradient 2) is an adenocarcinoma antigen expressed by many solid tumor types. In prostate, AGR2 is overexpressed in cancer cells compared with normal luminal cells, and a majority of primary prostate tumors are AGR2 positive [1], This pattern is similarly found in pancreatic [2, 3], oral [4], and breast cancers [5]. AGR2 is also highly expressed in non-small cell lung cancer (NSCLC) where high expression is associated with poor survival [6].

We have previously demonstrated the expression and role of AGR2 in initiation of pancreatic cancer [7], and that premalignant lesions of both the prostate and pancreas show AGR2 expression [3, 8]. AGR2 also plays a role in cancer spread [3, 4, 9]. Bone and soft tissue metastases in advanced prostate cancer show high AGR2 expression [10], and in pancreatic cancer, metastatic growth could be inhibited by targeting AGR2 [11]. Therefore, due to almost ubiquitous expression in solid tumors, expression in premalignant lesions and its involvement in metastatic disease, AGR2 represents an attractive target for cancer therapy. Importantly, while it is localized to the endoplasmic reticulum (intracellular iAGR2 [12]) in normal cells, AGR2 is secreted and found on the surface (extracellular eAGR2) of cancer cells [2, 8, 13–16]. Normal cells would thus likely be unaffected by anti(α)-AGR2 reagents. This property makes AGR2 a unique tumor-associated antigen (TAA).

One of the successful approaches in cancer therapy that targets TAA is the use of anti-TAA monoclonal antibodies and their engineered derivatives. For example, Herceptin (trastuzumab) is a well-known humanized monoclonal antibody used in the treatment of HER2-positive cancers [17], and Erbitux (cetuximab), a pan-erbB inhibitor, is used for cancers expressing different levels and subclasses of EGFRs [17] including NSCLC [18]. Kadcyla (trastuzumab emtansine, ɑ-HER2, -EGFR, -CD340) and Adcetris (brentuximab vedotin, ɑ-CD30) are examples of antibody-drug conjugates (ADC) approved to treat recurrent breast cancer or some hematological malignancies, respectively. Gemtuzumab ozogamicin (ɑ-CD33) is a recombinant humanized IgG4κ antibody, which is conjugated to a cytotoxic antitumor antibiotic directed against the CD33 antigen present on leukemic myeloblasts in acute myeloid leukemia [19]. All of these antibodies have received federal approval, and are used as effective cancer therapeutics. Recently, an ADC ɑ-PSMA was shown to produce clinically relevant decline in prostate-specific antigen (PSA) and circulating tumor cell counts in metastatic castration resistant prostate cancer [20]. However, lack of widespread application of many ADC treatments can be attributed to surface expression of these TAA in normal cells [21–23] leading to unavoidable side effects.

For clinical application, mouse antibodies are converted to human:mouse chimeric or humanized forms to reduce anti-mouse response; more importantly, mouse antibodies interact poorly with human effector cells and protein factors to mediate cell killing. Therapeutic antibodies promote target cell death through antibody-dependent cell-mediated cytotoxicity (ADCC), which is effected by immune cells through F_c_γR binding [24], while complement-dependent cytotoxicity (CDC) is triggered by binding of complement proteins to the F_c_ domain to form an attack complex [25]. In our previous work, radiolabeled cancer cells were exposed to antibodies and human serum or peripheral blood leukocytes [26, 27]. In CDC, the chimeric antibodies generated higher response at all complement titers than the mouse antibodies. In ADCC, the chimeric antibodies lysed a greater fraction of cancer cells at lower concentrations. Cell killing was specific because ADCC was not observed with cells lacking the targeted antigen [26, 27].

Using recombinant AGR2 as immunogen, we generated two ɑ-AGR2 monoclonal antibodies: P3A5 (IgG2a) and P1G4 (IgG1) [13]. We used several assays to ensure they recognize the native antigen [13]. P1G4 and P3A5 in ELISA could detect cancer-secreted AGR2 in voided urine from 1.26 to 181 pg/mL in a cohort of samples [13]. A commercial diagnostic company, MagArray, showed experimentally that our antibody pair detected AGR2 with better sensitivity than any other available antibodies (Dr. Heng Yu, personal communication). Both of these monoclonal antibodies (mAb) were therefore selected for humanization. Our study demonstrates the possibility that these ɑ-AGR2 reagents could be clinically useful in treating eAGR2^+^ cancer while sparing iAGR2^+^ healthy cells.

## RESULTS

### Cancer cell surface expression of AGR2

**Figure 1:**
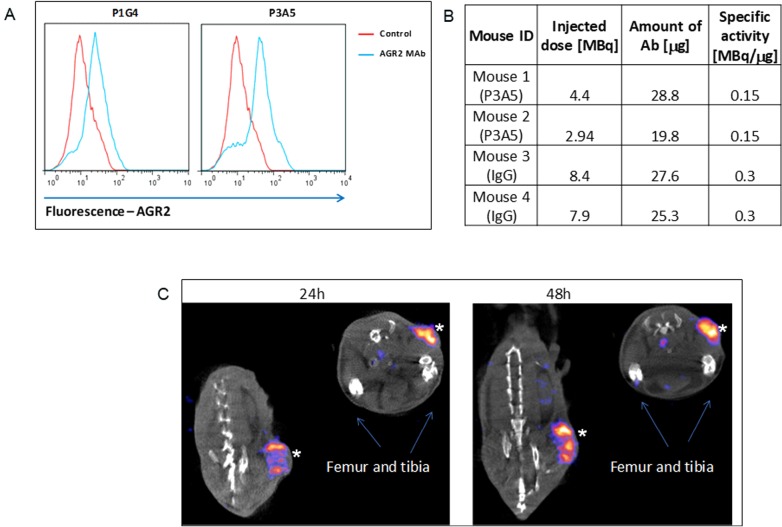
Cell surface expression of AGR2. **(A)** Fluorescence shifts of P1G4- and P3A5-labeled DT6606 primary pancreatic cancer cells (*vs*. control IgG) indicate that eAgr2 is present on the cell surface. Two cytograms are shown. **(B)** The table shows labeling of antibodies. **(C)** The representative images show DT6606 tumor uptake of radiolabeled P3A5 (marked by ^*^) at 24 h and 48 h post-injection. No significant label could be detected in iAgr2-positive normal tissues.

We have previously demonstrated the surface expression of AGR2 on human breast and pancreatic cancer cells [3]. Figure 1A shows cell surface detection by flow cytometry of Agr2 on DT6606 mouse pancreatic cancer cells using P3A5 and P1G4 antibodies. These cells were then used for tumor localization *in vivo*.

### Localization of ɑ-AGR2 mAb in mice implanted with Agr2^+^ tumors

Figure 1B shows the efficiency of radiolabeling, injected radioactivity values for P3A5 and control IgG (specific activity of radiolabeled P1G4 was suboptimal, and was not used). Images in Figure 1C of the implanted DT6606 tumor in syngeneic C57BL/6 mice show specific uptake of P3A5 from 24 to 48 hours post injection.

### Tumor growth inhibition by ɑ-AGR2

**Figure 2:**
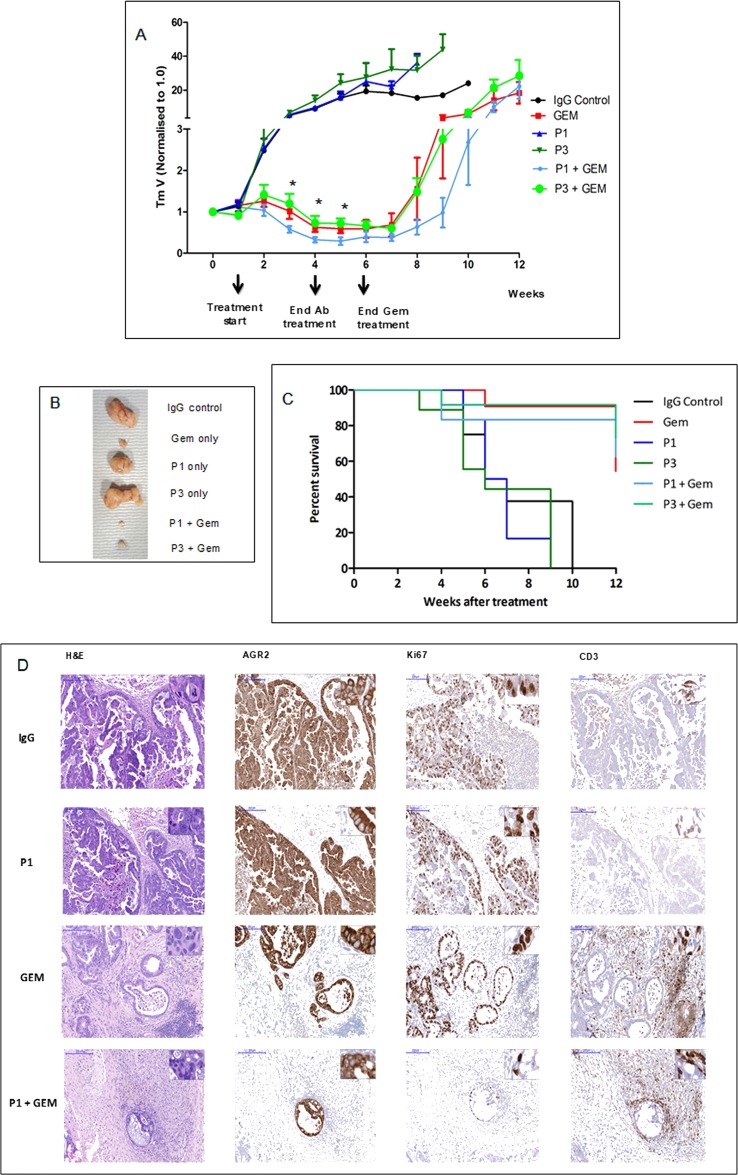
Tumor growth inhibition by drug in combination with antibodies. **(A)** PDX tumor growth in mice in response to treatment with ɑ-AGR2 P1 (P1G4) and P3 (P3A5), alone or in combination with Gem demonstrate that antibodies alone produced minimal effect; the best tumor suppression was achieved in the P1 + Gem group. Tumor volume is indicated on the *y*-axis, and treatment duration in weeks on the *x*-axis. Differences in tumor volumes between P1 + Gem *vs*. Gem were statistically significant (^*^) at weeks 3-5. **(B)** Tumors extracted at week 6 from the different groups are compared. **(C)** The Kaplan–Meier plot shows the percentage survival (*y*-axis) of mice across all six groups (in weeks on the *x*-axis). **(D)** Representative immunohistochemistry images on tumor tissue samples from IgG control, P1, Gem and P1 + Gem confirm expression of AGR2 in all tumors. Ki67 staining indicated that the tumors treated with Gem still had high proliferation rate, which was limited in P1 + Gem-treated tumors. The tumors from Gem- and P1 + Gem-treated mice exhibited abundant CD3^+^ T cell infiltration. Size bars = 200 μm. Inserts at the top right of individual images are at 200x magnification.

The *in vivo* anti-tumor activity of P1G4 and P3A5 antibodies was assessed using patient-derived pancreatic cancer xenograft (PDX) models. Tumor-bearing mice were treated with P1G4 (P1) or P3A5 (P3) alone or in combination with standard of care drug Gemcitabine (Gem) up to week 6 *vs.* Gem alone (Figure 2A). Antibody monotherapy showed no anti-tumor activity in the immune compromised hosts. Combination of P3A5 with Gem showed no additive activity compared to Gem alone. However, combination of P1G4 with Gem showed a stronger reduction in tumor volume as well as delayed tumor regrowth compared to Gem alone, even several weeks after discontinuation of treatment (Figure 2A). Figure 2B shows the tumors extracted from mice culled at 6 weeks due to tumor sizes reaching the maximum threshold (1 cm^3^) in the IgG, P1 and P3 groups. Figure 2C shows the percentage survival of mice in different treatment groups. Notably, by week 12, three of five mice in Gem-only group reached the tumor burden threshold, while this was the case in only one of four remaining P1 + Gem mice.

The anti-tumor activity of P1G4 in combination with Gem was confirmed in a follow up experiment (data not shown). Figure 2D shows the immunohistochemistry results: AGR2 immunoreactivity confirmed that all tumors were AGR2^+^. Ki67 staining indicated less proliferation in the P1G4 + Gem-treated tumors. An abundant infiltration of T cells was observed in tumors treated with Gem or P1G4 + Gem. No visible histological changes were detected in the internal organs (liver, spleen, stomach, intestine, colon, and pancreas).

### Generation of human:mouse chimeric P3A5 and P1G4 antibodies

**Figure 3:**
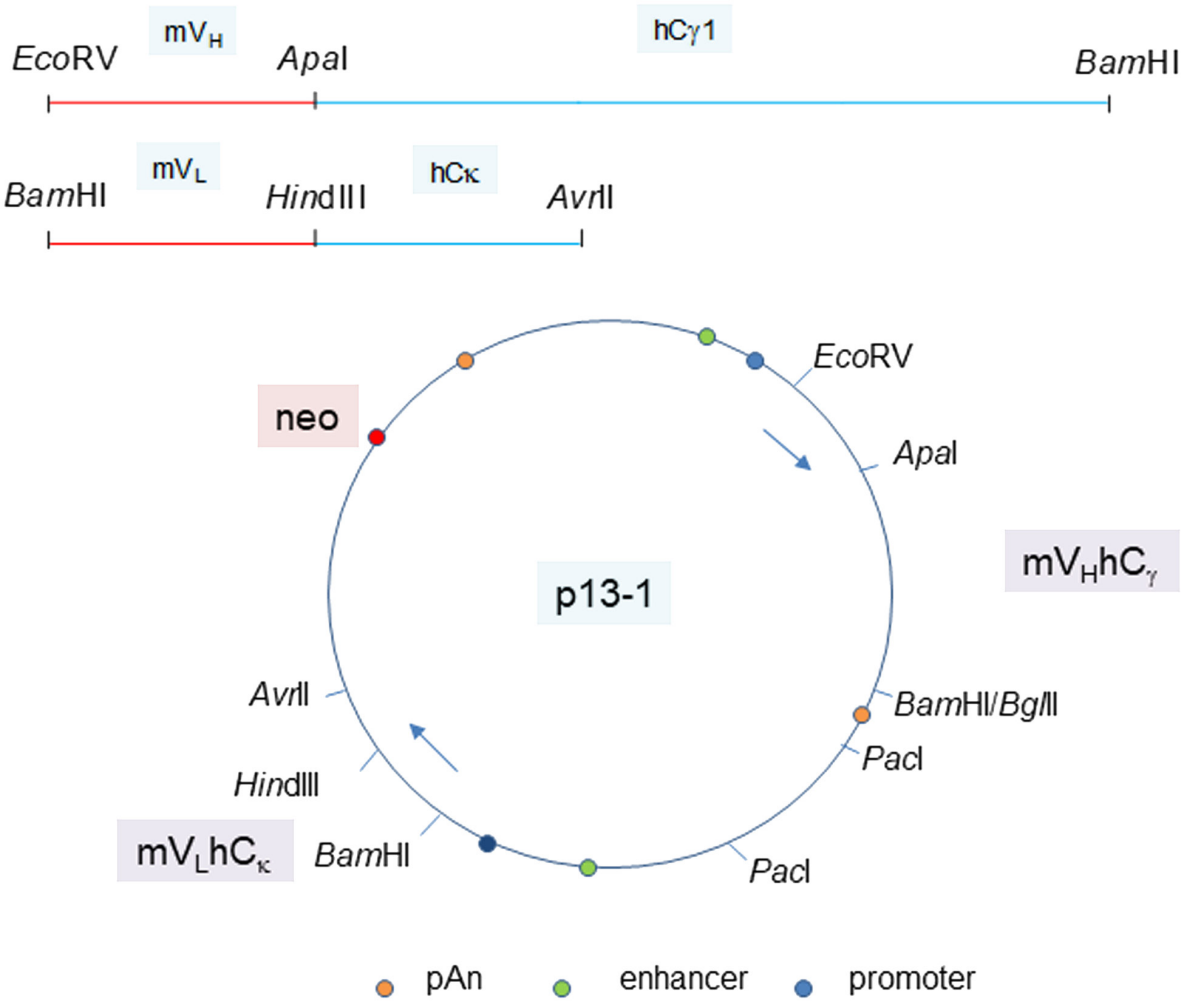
Plasmid vector for P3A5 H and L chain expression. The joined mouse V_H_ module (EcoRV-ApaI) and human C_γ1_ module (ApaI-BamHI), as well as the joined mouse V_L_ module (BamHI-HindIII) and human C_κ_ module (HindIII-AvrII) are shown. The H and L chain genes are cloned in the orientation depicted in plasmid p13-1. Gene expression control elements are identified. PacI is used to linearize the plasmid for transfection. Plasmid p20-6 has bsr instead of neo. Plasmids p40-1 and p50-1 (not shown) are the corresponding neo and bsr vectors for chimeric P1G4.

**Figure 4:**
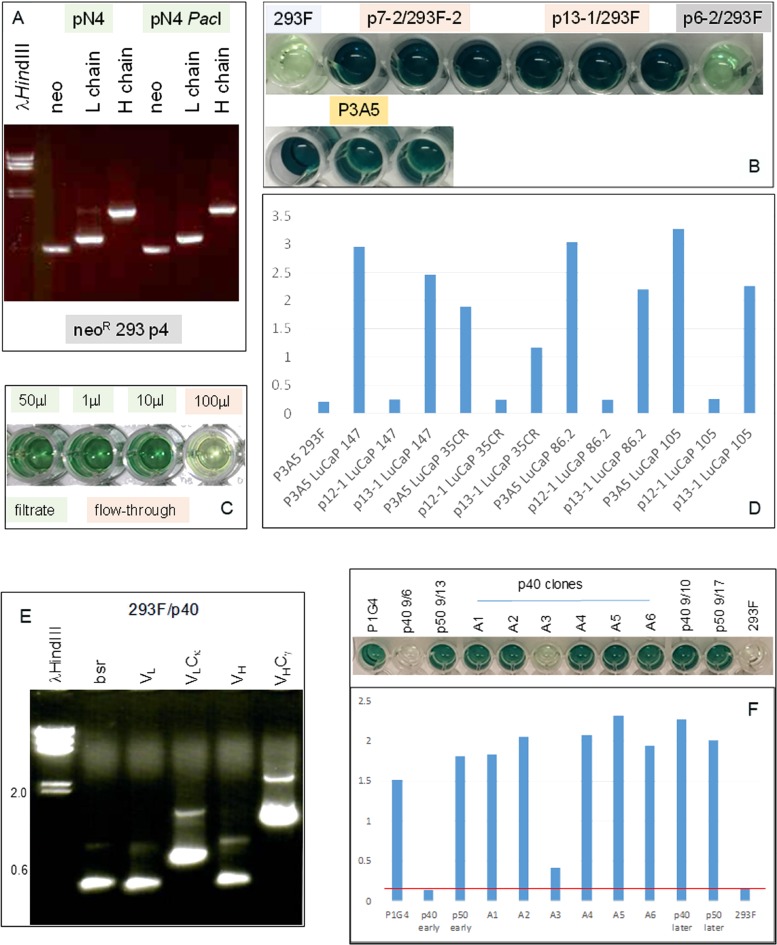
Immunoglobulin expression in transfected 293F cells. **(A)** RT-PCR analysis for neo (560 bp), L (720 bp) and H (1420 bp) chains in cells transfected with plasmid pN4 at passage 4 (p4) to show that the transgenes were being transcribed at equivalent levels. **(B)** ELISA result shows AGR2 binding by IgG1 from transfected 293F cells (in triplicate). The color intensity indicates similar binding affinity between the IgG1 constructs p7-2 and p13-1 *vs*. P3A5. Background consists of untransfected 293F and p6-2, which is a non-productive construct. Detection is by either HRP anti-human IgG for the transfected cell media or HRP anti-mouse IgG2a for P3A5. **(C)** ELISA shows IgG isolation by spin filtration. Background level was detected in the 100 μL flow-through well (4^th^) *vs*. the three wells (1^st^ – 3^rd^) containing different amounts of the filtrate/retentate. **(D)** Histogram representation of ELISA data shows comparable binding between 293F-synthesized human IgG and P3A5 in tissue digestion media of LuCaP lines (147, 35CR, 86.2, 105) with different levels of AGR2 expression. p12-1 is a defective construct, and served as negative control. Absorbance units are indicated on the *y*-axis. **(E)** RT-PCR analysis of 293F/p40-1 P1G4 chimeric detected transcripts for bsr (420 bp), V_L_, V_L_C_κ_, V_H_, and V_H_C_γ1_. **(F)** The histogram bars show AGR2 binding activities detected in media supernatant of p40, p50, and p40 clones A1-A6 (absorbance units on the *y*-axis). The positive control is mouse P1G4 and the negative control 293F cells. “Early” and “later” indicate media harvested during early and late passages, respectively, of cultures after drug selection.

The human C-domain modules C_γ_ and C_κ_ were joined to the mouse V_H_ and V_L_ modules in a single plasmid vector (Figure 3). Reverse transcriptase-polymerase chain reaction (RT-PCR) of cells transfected with plasmid pN4 showed equivalent transcript levels for neo (encoding G418 resistance), L chain and H chain (Figure 4A). The neo and L chain were transcribed from one promoter, while the H chain from another. The in-frame ATG before the start codon in the P3A5 mouse V_L_ proved inhibitory for translation as no secreted immunoglobulin was detected in the culture media of pN4. A primer containing a canonical Kozak sequence was used to replace that part of the 5’ noncoding segment (p7-2). The same sequence was also used for the V_H_ module (p13-1). This proved successful (see below). There was no notable difference in protein expression level for the H chain with either the native or the artificial Kozak sequence.

### Expression of functional IgG by transfected cells

The cell-free culture media supernatant was assayed for ɑ-AGR2 activity by ELISA. Figure 4B showed that the chimeric IgG1 antibodies produced by plasmids p7-2 and p13-1 were similar to P3A5 in AGR2 binding as indicated by the color intensity of the chromogen detector. For comparison, the negative controls were 293F media and media of p6-2 with a defective L construct. Media from culture passages showed that immunoglobulin synthesis continued indicating integration of the transgenes into the host genome. G418 selection was no longer required, therefore this toxic compound is not necessary in IgG preparations for clinical use. ELISA (Figure 4C) showed that spin filtration was effective in concentrating the immunoglobulins (150 kDa). In addition, chimeric IgG1, like P3A5, detected similarly different levels of AGR2 produced by several prostate cancer PDX LuCaP lines (Figure 4D).

AGR2 binding activity of chimeric IgG2 (p16-1) and IgG4 (p17-1) was also demonstrated by ELISA (Supplementary Figure 1). Several clones of either isotype tested showed similar levels of activity. The IgG3 isotype was not obtained as the C_γ3_ sequence was not detected in the white blood cell cDNA used for cloning IgG genes.

The P1G4 constructs, p40-1 and p50-1, were also successful in producing the corresponding IgG1 transcripts in 293F cells (Figure 4E). The P1G4 chimeric IgG1 showed similar AGR2 binding activity as P1G4 (Figure 4F).

### Increased IgG production of bsr^R^/neo^R^ cells

**Figure 5:**
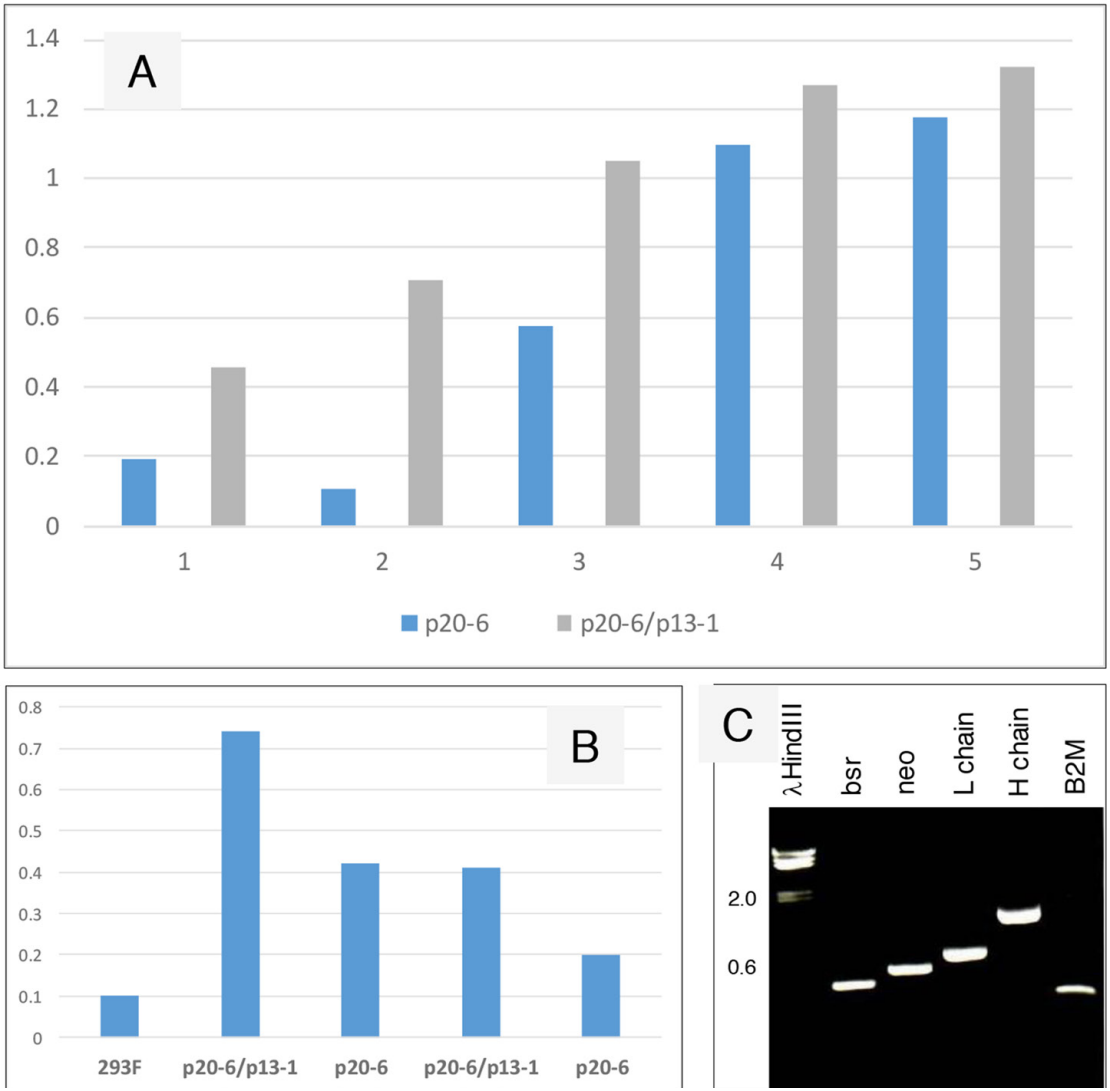
Increased IgG production from serially transfected 293F cells. **(A)** Cell-free media supernatant from increasing amounts of cells seeded in culture wells were assayed for AGR2-binding activity. The source of AGR2 was provided by LNCaP cells transfected with AGR2 (based on the same plasmid vector). Absorbance units are shown on the *y*-axis. **(B)** The volumes of media supernatant obtained from similar numbers of cells were assayed. The negative control is media from untransfected 293F cells. Absorbance units are shown on the *y*-axis. **(C)** Transcript levels of bsr, neo, L and H chains, B2M were detected in the doubly transfected clone A3.

Figure 5 compares production of human IgG by 293F cells transfected with p20-6 (bsr^R^) and cells transfected with p20-6 followed by p13-1 (neo^R^). The resultant double drug resistant cells were shown positive by RT-PCR for *bsr* and *neo*. Similar numbers of cells were plated in 6-well plates, and the cell-free media was collected after 3 days. ELISA showed that more AGR2-binding activity was observed in p20-6/p13-1 than p20-6. AGR2-binding activity was higher in p20-6/p13-1 than p20-6 when 10 μL and 1 μL of cell-free media from cultures containing 10^6^ cells were compared.

### Anti-tumor effect of chimeric antibodies on PC3 prostate cancer cells

**Figure 6:**
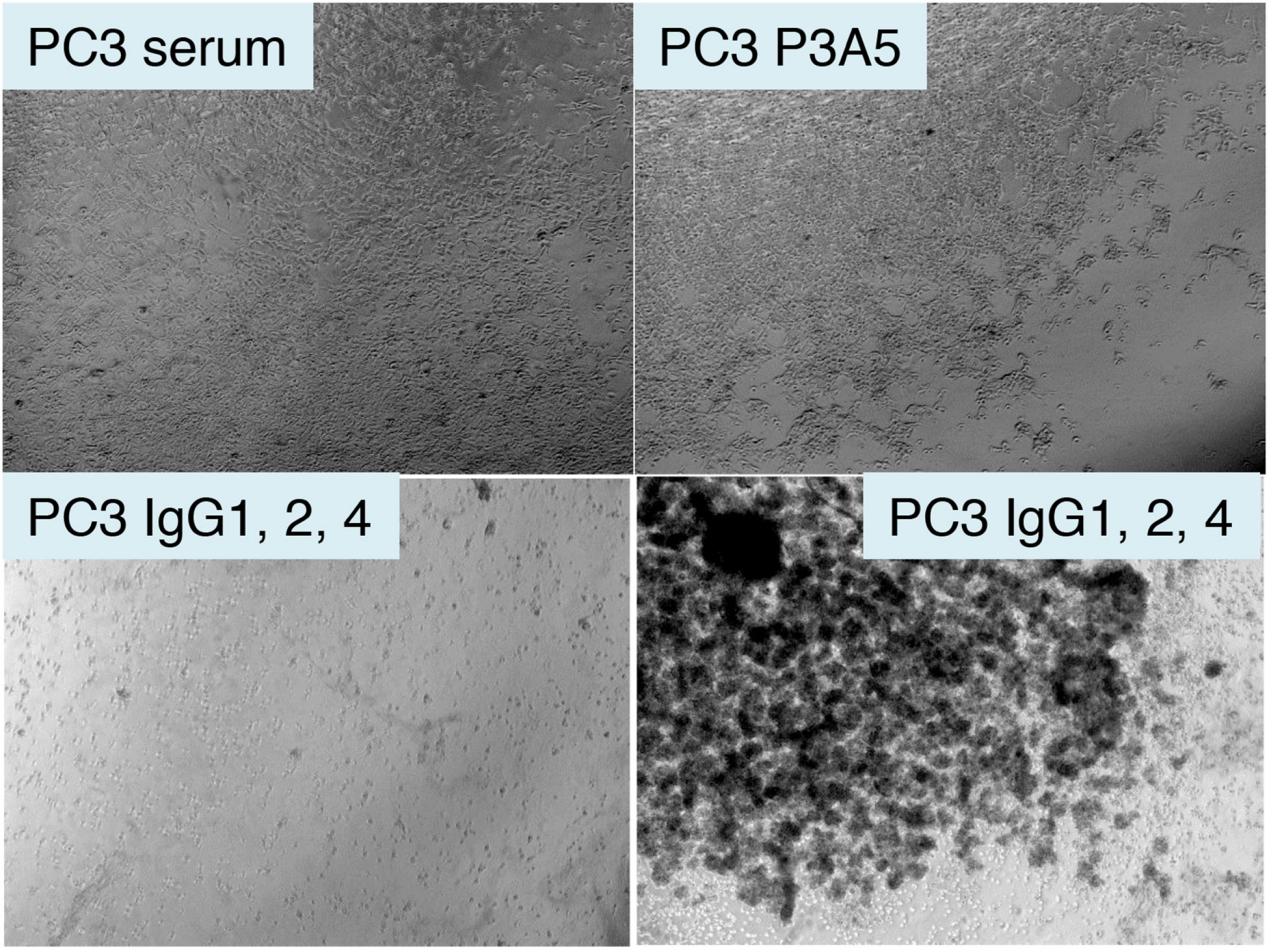
Treatment of AGR2^+^ PC3 cells by chimeric antibodies. Shown are photomicrographs of plated eAGR2^+^ PC3 cells incubated in human serum (top left); serum with P3A5 (top right); serum with IgG1, IgG2, IgG4 (bottom left and right).

Spin concentrated chimeric IgG was used on eAGR2^+^ PC3 cells with serum donated from healthy volunteers. Cells plated in culture wells were incubated in human serum with added AGR2 antibodies. Figure 6 shows that in the well with human serum alone, there was no observable effect on cell viability. Likewise, no effect was seen in the well with mouse P3A5. In the well with chimeric γ1, γ2, and γ4, cell growth was inhibited as indicated by large surface areas of the well devoid of cells while clusters of pyknotic cells were seen in suspension after 3 days.

## DISCUSSION

Due to frequent expression in common solid tumors, expression in premalignant lesions, circulating tumor cells and metastases [3-8], AGR2 represents an exciting cancer-specific target. In tumor localization experiments, we demonstrated the accumulation of radiolabeled mAb in the pancreatic tumors. The fortuitous cross-reactivity between human AGR2 and mouse Agr2 suggests that a similar result could be obtained in pancreatic cancer patients with minimal targeting of iAGR2-expressing healthy cells. Furthermore, since the generated antibodies could recognize murine Agr2, one would expect severe deleterious effects on multiple organs with abundant circulating ɑ-AGR2/Agr2 after treatment. However, this was not observed and the internal organs examined showed no histological evidence of toxicity, as did the organs of mice in the production of hybridomas [13].

The combination of anti-cancer drugs and antibodies has been shown to be clinically useful in treating lung cancer [28]. At present, we do not know the precise mechanism in the enhancement of Gemcitabine inhibition of tumor growth by P1G4 mAb, and the reason why P3A5 was not as efficient. This could be related to the different epitopes recognized by the antibodies, need for increased therapeutic doses or a longer treatment regimen. Additionally, whether drug + other TAA antibodies would produce a similar or improved effect in the pre-clinical pancreatic cancer model remains to be determined. Interestingly, we observed CD3^+^ lymphocytes in our treated mice, which was unexpected. It is likely that these could be extrathymic T cells, as athymic nude mice carry neither conventional T cells nor NKT cells of thymic origin. However, NK1.1-TCRint cells of extrathymic origin can be found in the liver and other immune organs, and may also infiltrate tumors [29].

A humanized ɑ-AGR2 (18A4Hu) and its murine version (18A4) alone were reported to have inhibitory effect on AGR2^+^ ovarian cancer xenograft SK-OV-3 [30]. This result is somewhat perplexing (although not discussed in the paper) because in nude mice with no immunity, antibodies by themselves would have no significant effect on xenograft growth as shown by our mAb in the absence of Gem.

In prostate cancer, cancer-associated antigens PSA, PSMA, PAP, PSCA, MUC1 and PAGE/GAGE have been used to stimulate T cell-mediated immunity [31]. Lack of consistent success could be partly due to some of these antigens being secreted molecules or not prostate-specific [21, 32]. In contrast, AGR2 expression differs between cancer and normal cells in subcellular localization, eAGR2 *vs*. iAGR2. We have previously shown that normal cells in lung and bladder do not secrete AGR2 as we found only background levels of AGR2 in serum samples using sensitive targeted proteomics [33]. A somewhat contrary result reported significant amounts of AGR2 in blood, but these were based on the use of polyclonal AGR2 antibodies [34], and have not been validated by multiple large-scale proteomics studies (archived in *PeptideAtlas*). Furthermore, normal urine contains little AGR2 even though the entire urothelium is positive for AGR2 [16]. This research did not set out to quantify the percentage of cancer-secreted AGR2 that might be targeted by ɑ-AGR2. However, it is an important factor that should be established in future studies.

In addition to pancreatic cancer, the lysis of eAGR2^+^ PC3 cancer cells *in vitro* shows that targeting AGR2 could also prove to be effective against prostate cancer. The common expression of AGR2 in other solid tumors suggests that such treatments may also be effective in other cancer types, and in the orthotopic setting, but these still remain to be established. Similarly, the anti-metastatic activity of ɑ-AGR2, as recently shown in the lung metastasis mouse model [35], warrants further investigation. Cancer-specific cell surface expression of eAGR2 makes cancer vaccination an exciting possibility, where a primed immune response could potentially eliminate any newly arisen eAGR2^+^ cancer cells.

The pVITRO plasmid and 293F cells proved to be a good system for immunoglobulin synthesis given that both L and H chains can be inserted in one vector. In the transfected cells, the L and H chain proteins were correctly processed, assembled, and secreted. The IgG molecules can be readily concentrated by spin filtration after growth in serum-free media. For protein expression, only the Kozak box outside the coding region is sufficient. The native Kozak sequences are as efficient as the canonical sequence. The strategy of serial transfection using different drug selection was successful in enhancing the yield of antibodies. A third plasmid with hygromycin selection might increase production even further, and economically, it is fairly inexpensive to obtain antibodies from transfected 293F cells. The chimeric IgG1 and IgG2 isotypes can substitute for the murine IgG1 (P1G4) and IgG2a (P3A5) in ELISA. We are now in the process of cloning the human IgG3 to complete the human gamma immunoglobulin repertoire [36]. As P3A5 and P1G4 bind to different epitopes, they might also act synergistically, which also remains to be determined.

In summary, we present further evidence that AGR2 is a promising therapeutic target and report on the production of two humanized antibodies that could, after further evaluation, be employed for therapeutic purposes.

## MATERIALS AND METHODS

### Flow analysis of AGR2 cell surface expression

Mouse ɑ-AGR2 monoclonals P1G4 and P3A5 were purified from growth media of their respective hybridoma clones [13]. The mouse Agr2^+^ primary pancreatic cancer cell line DT6606 (PdxCre/LSL-*Kras*^G12D^) [37, 38], were harvested by trypsin/EDTA, resuspended in DMEM, 0.1% BSA, 0.1% sodium azide and incubated with antibodies on ice 45 min. Bound antibodies were detected with dye-conjugated anti-mouse IgG2a. Labeled cells were scanned on a BD FACSAria II Cell Sorter (BD Biosciences, San Jose, CA) and analyzed using CellQuest Pro software (BD Biosciences).

### Antibody labeling and biodistribution

The antibodies were conjugated on their lysine residues using p-SCN-Bn-CHX-A”-DTPA (Macrocyclics, Plano, TX, USA) [39]. Briefly, the antibodies were dialyzed against 1L 0.05 M HEPES, pH 8.5 containing 5 g Chelex 100 (Bio-Rad, Hercules, CA, USA) using Maxi GeBAflex-tube dialysis tubes (MWCO 8 kDa, Gene Bio-Application, Israel). CHX-DTPA was then added in 5-fold molar excess and reacted for 2 h at room temperature. Conjugation solutions were purified by dialysis into 0.4 M ammonium acetate pH 5.5. ^111^In radiolabeling was carried out at 37° for 30 min at pH 5, and analysed by iTLC-SG (Agilent, Santa Clara, CA, USA). For tumor localization, C57BL/6 mice carrying DT6606 tumors were injected i.v. with radiolabeled antibodies and imaged at 24 and 48 h post injection by SPECT/CT (Bioscan, Santa Barbara, CA, USA.

### Tumor growth inhibition

For *in vivo* testing, CD1 female mice were implanted with pancreatic PDX (6 groups of 6 mice each) subcutaneously in both flanks as described previously [40]. Each animal was treated by intraperitoneal injection of antibodies at 5 mg/kg of body weight twice a week for four weeks; Gemcitabine (G6423 Gemcitabine hydrochloride >98% HPLC, Sigma, St. Louis, MO, USA) was administered at 100 mg/kg once a week for 6 weeks. Six experimental groups were: 1. IgG control; 2. Gem only; 3. P1 only; 4. P3 only; 5. P1 + Gem; 6. P3 + Gem. Tumors were measured at implantation, and then once every week, and animal weights recorded twice a week. After treatment cessation, mice were continuously monitored to week 12. After sacrifice, the liver, pancreas, spleen, lungs, stomach, intestine and colon were collected and examined histologically. All animal studies were performed in compliance with the UK Animals (Scientific Procedures) Act 1986 and the Code of Practice for the Housing and Care of Animals used in Scientific Procedures (Home Office, UK).

### Immunohistochemistry

For immunohistochemical analysis, tissue blocks were serially cut into 5 μm sections, which were first stained by hematoxylin and eosin (H&E) to assess histology, and then for AGR2 (C1313 Santa Cruz Biotech, Santa Cruz, CA, USA (1 = 30)), Ki67 (2746/1 Epitomics, Burlingame, CA, USA (1 = 6000)) and CD3 (A0452 Dako, Santa Clara, CA, USA 1 = 200)). Immunohistochemistry was performed following protocols for the Ventana Discovery System automated platform (Roche, Switzerland). Histology was reviewed using the digital program Pannoramic Viewer (3DHistech, Hungary).

### Human immunoglobulin cDNA

Blood samples were drawn from consented donors, and partitioned on density gradients. White blood cells were collected, lysed for RNA isolation and cDNA synthesis. The resultant cDNA was checked by PCR for B2M (β2-microglobulin). The human gamma heavy chain constant module was obtained by PCR with primers: Cγ-5 GGTCACCGTCTCTTCAGCCTCCACCAAGGGCCCATCG with ApaI restriction enzyme site and Cγ-3 GAATTC GGATCCTCATTTACCCGGAGACAGGGAGAGGCTCTTCTG. The human kappa light chain constant module was obtained by primers: Cκ-5 CTCACTTTCGGCGGAGGG ACCAAGCTTGAGATN with HindIII site and Cκ-3 GAATTCGGATCCCTAACACTCTCCCCTGTTGAAGCTCTTTGTGACG. The products were each inserted into plasmid pCR2.1 (Thermo Fisher, Waltham, MA, USA). DNA sequencing showed that γ1, γ2 and γ4 constant domains were cloned – both γ1 and γ4 have a SacII site, while γ4 has an EcoRI site, γ2 has no SacII or EcoRI.

### P3A5 and P1G4 mouse immunoglobulin cDNA

P3A5 and P1G4 hybridoma cells were lysed for cDNA synthesis. The heavy chain variable module (IghV1-37) was obtained by primers: P3A5 H-5 GTCCTCGGTACCGATATCGAACACACTGACTCCAACTATGGGATGGACGTGGATC and P3A5 H-3 CACACAGGGGCCAGTGGATAGACCGATGGGCCCTTTGTGCTGGCNNNN (with ApaI site). The light chain variable module (IgκV6-15) was obtained by primers P3A5 L-5 GAATTCGAAGATATCAGATCAGCATGGGCATCAAGATGGAGTCACAGACTCAG and P3A5 L-3 CAGCATCAGCCCGTTTTATTTCAAGCTTGGTCCC (with HindIII site). The primer sequences were based on previous DNA sequence analysis of P3A5 immunoglobulin cDNA. P3A5 mRNA was reverse transcribed with primers of mouse C_κ_ and C_γ2a_ matching sequences a short distance beyond the V/C junctions. The same primers were used for DNA sequencing of the cDNA products. The L-5 sequence contains an in-frame ATG upstream of the start codon (which could be due to a misread of DNA sequence data of the cDNA 5’ end). Primer L-5c GAATTCGAAGGATCCCAAGCCACCATGGAGTCACAGACTCAGGTCTTG was used to remove this ATG and introduce a consensus Kozak box (gccRccAUGG). Primer P3A5 H-5a GAATTCGAAGATATCCAAGCCACCATGGGATGGACGTGGATCTTTCTCTTC was used to introduce the canonical Kozak box to the heavy chain construct. Similarly, mouse C_κ_ and C_γ1_ primers were used to determine the V domain sequences of P1G4. Primers for cloning P1G4 V_H_ and V_κ_ were: heavy chain (IghV1-52) H-5 GAATTCGAAGATATCCAAGCCACCATGGGATGGAGCTGCATCATCCTC and H-3 GATGGGCCCTTTGTGCTGGCTGAGGAGACTGTGAGAGTGGTGCC; light chain (IgκV4-68) L-5 GAATTCGAAGGATCCCAAGCCACCATGGATTTTCAAGTGCAGATTTTC and L-3 CAGCATCAGCCCGTTTATTTCAAGCTTTGTCCCCGAGCCGAACGTG. The canonical Kozak box was used in both chains.

### Construction of expression plasmids

The mouse V_H_ and human C_H_ modules were joined at the *Apa*I site; the mouse V_L_ and human C_L_ modules were joined at the *Hin*dIII site. For mammalian cell expression, plasmid pVITRO1-neo-mcs (InvivoGen, San Diego, CA, USA) was used, which contains modules of SV40 and CMV enhancers, mEF1 and rEF1 promoters, SV40 transcription termination and poly-adenylation [41]. The chimeric heavy chain *Eco*RV-*Bam*H1 module was inserted into the *Eco*RV and *Bgl*II sites of mcs2, and the chimeric light chain *Bam*HI-*Avr*II module was inserted into the *Bam*HI and *Avr*II sites of mcs1 to obtain plasmid pN4 (γ1). Other constructs (see also Supplementary Materials) included p6-2 (L-5a, γ1), p7-2 (L-5c, γ1), p12-1 (L-5a, H-5a, γ1), p13-1 (L-5c, H-5a, γ1), p16-1 (L-5c, γ2), and p17-1 (L-5c, γ4). pVITRO1-blasti (InvivoGen) was used to replace the *neo* gene of p13-1 with the *bsr* (encoding blasticidin resistance) gene via *Xba*I/*Eco*RI digestion to produce plasmid p20-6. This construction was used to test whether serial transfection – p13-1 followed by p20-6 or p20-6 followed by p13-1 – would increase the yield of secreted IgG1 by the resultant neo^R^/bsr^R^ 293F cells.

P1G4 chimeric plasmids were constructed by replacing the P3A5 H and L chain modules in p20-6 with the respective P1G4 H and L chain modules: p40-1*bsr*^R^ (*Bam*HI-*Avr*II L module) and p50-1*bsr*^R^ (*Bgl*II/*Bam*HI-*Avr*II L module) (Supplementary Materials). Plasmids for IgG2, IgG4, and neo^R^ version were also generated.

### Gene transfection

Plasmid DNA linearized by *Pac*I was used in transfection of human embryonic kidney fibroblast HEK293F by electroporation (AMAXA nucleofactor program A-024, Lonza, Switzerland). At 2-3 days post-transfection, the cells were placed in media with 1 mg/mL G418 (Mediatech, Manassas, VA, USA) for selection. The transfected G418-resistant cells were weaned from FBS by serially culture from 10%, 5%, 2% to serum-free media (FreeStyle 293, Thermo Fisher). Cloning was accomplished by picking colonies with pipetor. RNA was isolated and analyzed by RT-PCR with primer pairs of mouse V 5’ and human C 3’ (see above) as well as neo5: GCAGCTGTGCTCGACGTTGTCACTG and neo3: CAGAGTCCCGCTCAGAAGAACTCGTC using “hot start” with 35 cycles of 94°, 30 s; 57°, 30 s; 72°, 60 s. For p20-6 transfection, blasticidin (Thermo Fisher) was used at 5 μg/mL. Both G418 and blasticidin concentrations were pre-determined. Primers for bsr were bsr5: ATGAAGACCTTCAACATCTCTCAGA and bsr3: TTAGTTCCTGGTGTACTTGAGGGG.

In serial transfection, 293F cells were first transfected by p20-6, and blasticidin resistant clones were selected. IgG-producing bsr^R^ cells were then transfected by p13-1, and cultured in G418. Cell-free media supernatants of the resultant cells: 293F/p20-6*bsr*^R^/p13-1*neo*^R^ were assayed for secreted human IgG1. The double drug resistant cells were grown on serum- and drug-free media for large-scale production.

### ELISA of chimeric antibodies

For P3A5, each well of 96-well plates was coated with 0.2 μg P1G4 in 100 μL PBS overnight followed by rinsing with PBS-0.05% Tween, and blocking with 1% BSA-PBS. Cell-free prostate LuCaP xenograft tissue collagenase digestion media containing secreted AGR2 [42, 43], diluted 1/10, was added. P3A5 (0.2 μg) or media (100 μL) of transfected 293F cells were used for detection, followed by goat anti-mouse IgG2a-HRP or goat anti-human IgG-HRP (Southern Biotech, Birmingham, AL, USA), where appropriate, diluted at 1:2000 in 1% BSA-PBS followed by 1-step ABTS (Thermo Fisher). Wells were scanned at λ = 415 nm after 5-30 min [13]. For P1G4, the wells were coated with P3A5. Goat anti-mouse IgG1-HRP for positive control and goat anti-human IgG-HRP were used for detection.

### Spin concentration of immunoglobulin

Positive transfected cells were grown in 100 mL serum-free media without drugs. The cell-free media were centrifuged in 15-mL aliquots through 30K spin cartridges (Millipore, Burlington, MA, USA) in 50 mM NH_4_HCO_3_, 4K, 60 min to <1 mL in volume. ELISA was used to measure IgG in the concentrate.

### *In vitro* testing of cancer cell lysis

For anti-tumor effect, eAGR2^+^ PC3 cells [13] were incubated with human serum plus antibodies. The cells were cultured in a 24-well plate till about 75% confluent. Human serum was used from fresh blood donations after centrifugation to remove blood cells, and 50 μL was added to the wells. Fifty μL each of spin concentrated P3A5 IgG1, IgG2, IgG4 were added for incubation at 37°. Photomicrographs of the culture were taken.

### Statistical analysis

The Mann-Whitney U test was used to compare the P1 + Gem combination group to Gem-only group. Statistical analysis was performed and graphs plotted using the GraphPad Prism software and *P* values of <0.05 were considered statistically significant.

## SUPPLEMENTARY MATERIALS FIGURE


