# Supplementary material for: AGR2, a unique tumor-associated antigen, is a promising candidate for antibody targeting
Source: Oncotarget. 2019 Jul 2;10(42):4276–89. doi: 10.18632/oncotarget.26945 (PMC6611513; doi:10.18632/oncotarget.26945)
Supplement: Supplementary file 1 [file oncotarget-10-4276-s001.pdf]

AGR2, a unique tumor-associated antigen, is a promising candidate for antibody targeting

SUPPLEMENTARY MATERIALS

For the L chain of P3A5, a third construction was tested. Primer L-5a GAATTCGAAGGATCCCAAGCC ACCATGGATTTTCAAGTGCAGATTTTCAGCTTC CTGCTAATCAGTGCTTCAGTCATAATGTCCAGA GGAGAGATTCTGATGACCCAGTCTC (synthesized in two halves) was used to incorporate the leader of a different light chain variable sequence (found in at least eight archived mouse Vκ *vs*. four of P3A5 Vκ, NCBI database) that was known to be expressed in transfected mammalian cells [26]. This construct in plasmids p6-2 and p12-1, however, did not produce any immunoglobulin in transfected cells. The leader sequence might be incompatible in post-translational processing or folding. For the L chain of P1G4, a second L-5 primer with *Bgl*II instead of *Bam*HI was used for cloning to account for a gene-internal *Bam*HI site. Instead of a four DNA fragment-ligation to generate plasmid p40-1, a three DNA fragment-ligation was done to generate plasmid p50-1.

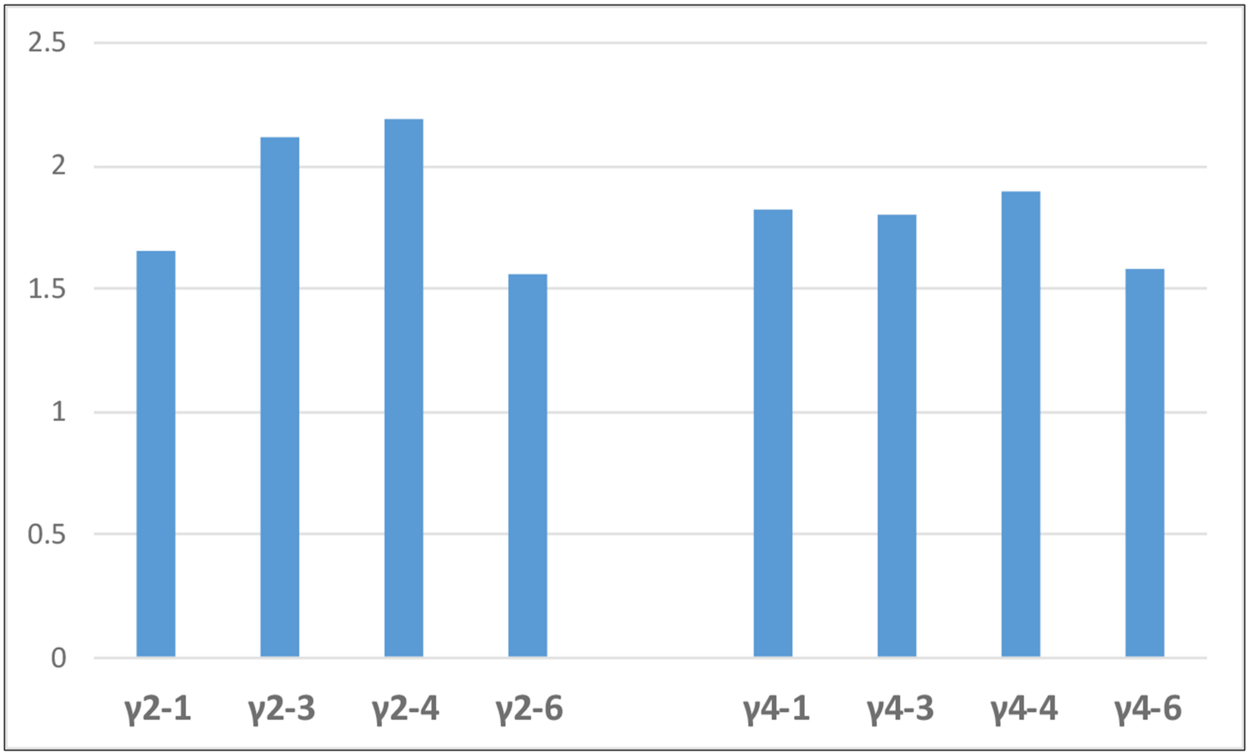

Supplementary Figure 1: AGR2 binding by IgG2 and IgG4 isotypes. The histogram shows ELISA data of four IgG2 clones (left) and four IgG4 clones (right). Absorbance units are shown on the *y*-axis.
